# Humination Modification: A Green Approach to Improve the Material Properties of Scots Pine (*Pinus sylvestris* L.) Sapwood

**DOI:** 10.1021/acsomega.4c09540

**Published:** 2025-01-15

**Authors:** Amir Ghavidel, Arantxa Eceiza, Xinfeng Xie, Reza Hosseinpourpia

**Affiliations:** † School of Engineering, University of Northern British Columbia, 499 George Street, Prince George V2L1R7, British Columbia, Canada; ‡ Materials + Technologies’ Group, Chemical & Environmental Engineering Department, Polytechnic College of San Sebastian, University of the Basque Country UPV/EHU, Pza. Europa 1, 20018 Donostia-San Sebastián, Spain; § College of Forest Resources and Environmental Science, Michigan Technological University, Houghton, Michigan 49931, United States; ∥ Department of Forestry and Wood Technology, Linnaeus University, Lückligs Plats 1, 35195 Växjö, Sweden

## Abstract

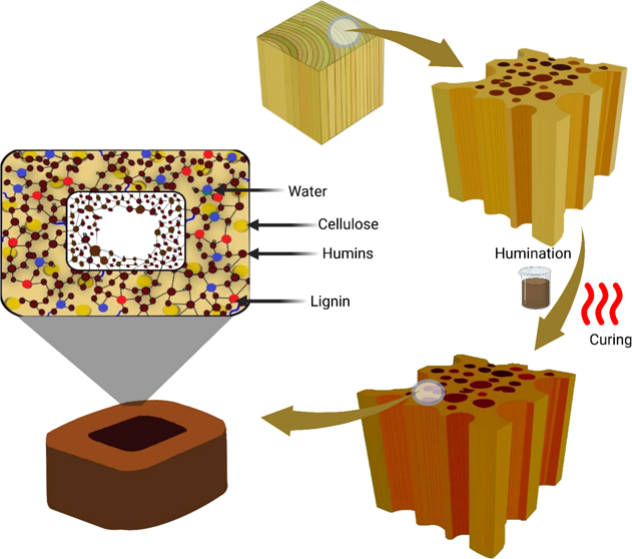

Recently, wood modification with environmentally friendly modification agents has received special attention. To this end, this study was conducted to use humin fractions, in combination with citric acid (CA) and succinic acid (SA), as reaction catalysts for the modification of Scots pine (*Pinus sylvestris* L.) sapwood. The effects of humination modification were evaluated by means of dimensional stability, static and dynamic mechanical properties, thermal stability, crystalline structure, and biological durability tests on modified samples and compared with the unmodified reference ones. According to the results, the dimensional stability of the huminated samples significantly increased, and this increase with the presence of catalysts was higher than the sole humin-modified samples. The static mechanical properties were considerably improved by 17–24% in the modulus of rupture (MOR) and by 11–12% in the modulus of elasticity (MOE). An apparent increase in the storage modulus of huminated wood was also determined by dynamic mechanical analysis (DMA). Although the thermal degradation of the samples was slightly shifted to lower temperatures after humination, the modification effect was more pronounced on the residual mass retention compared to the unmodified samples. The biological durability against white and brown rot fungi was also significantly improved by the humination modification. Overall, the humination modification showed huge potential as a green approach to enhance the wood properties for outdoor applications.

## Introduction

Wood is one of the most important renewable materials that has been widely used in many ways in our lives. As a natural material, it is prone to degradation by natural agents; thus, for long-term use, it needs to be protected to provide resistance to biotic and abiotic agents.^1,2^ Recent attention has been directed toward employing environmentally friendly approaches for wood protection. Besides the well-known industrially established thermal modification process, using biobased chemicals to improve the material properties of wood, such as natural oils, polyphenols, or even side streams from agricultural and forestry industrial processes, has received wide attention recently. Tung oil polymerizes to form a waterproof covering deep within the wood fibers, which reduces the degree of absorbed or desorbed moisture, hence avoiding size fluctuations; it resists decay by inhibiting the growth of fungi and bacteria at the wood fiber interfaces. On the other hand, tannins chemically interact with wood fibers to form complexes that enhance resistance to attacks by microorganisms and, hence, structural strength. Tannins bind with cellulose and lignin, thus toughening the wood so it warps or cracks less over time.^3,4^ Very recently, Ahmed and colleagues^5^ quoted that the water-related properties of Scots pine sapwood were highly improved by tannin modification. The leaching stability of tannin-based formulas in wood cells was also strongly improved by biobased cross-linking agents.

Humins are heterogeneous and polydisperse macromolecules mostly made of furanic rings with various functional groups, such as aldehydes, carboxylic acids, and hydroxyls.^6−8^ It is produced from the methanolic cyclodehydration of d-fructose and formed when an acid catalyst, traditionally sulfuric acid, is present in the process. It is a highly viscose tar-shaped syrup, and its chemical composition depends mainly on reaction conditions, residence time, and the type of carbohydrate feedstock.^9^ Water-stable humin-based matrices form through catalytic processes that involve polymerization and stabilization mechanisms. Acidic catalysts facilitate the creation of water-stable humin matrices through esterification.^10,11^ Humins have been employed previously to improve the properties of wood. The first investigation was carried out by Sangregorio and co-workers^12^ by comparing the huminated with furfurylated Scots pine samples. The authors reported comparable dimensional stability in huminated wood to that of furfurylated ones. The thermomechanical properties of furfurylated and huminated samples determined by dynamic mechanical analysis (DMA) showed an identical storage modulus at temperatures below 75 °C, which was higher than that of untreated wood, and then a sharp decrease in huminated wood at temperatures above 100 °C. This was followed by a considerably higher tan δ of huminated samples than furfurylated and untreated wood at temperatures above 120 °C, indicating a substantial change in the mechanical properties of huminated wood at elevated temperatures. Moreover, huminated wood exhibited higher fire resistance properties, like a longer ignition time, a slower heat release rate (decreased by 13%), and lower CO formation, compared with unmodified and furfurylated wood. Ghavidel and Hosseinpourpia^11^ reported that the humination of Scots pine sapwood microveneers in the presence of acidic catalysts significantly contributes to the enhancement of the photodegradation stability of the microveneers as compared to the unmodified ones. They found that huminated microveneers retained significant mass after 144 h of exposure to the accelerated artificial weathering test compared to unmodified samples. Although the microtensile strength of the samples significantly declined following exposure to artificial weathering, the huminated microveneers using acidic catalysts showed considerable strength retention as compared with the sole huminated and unmodified samples. While the potential of humination modification to improve the properties of wood is apparent from the studies mentioned above, more research on solid wood is required before moving to the industrial process. This study was thus aimed to explore the effect of humination modification on dimensional stability, e.g., antiswelling efficiency (ASE), maximum swelling, and bulking and mechanical properties, e.g., modulus of rupture (MOR), modulus of elasticity (MOE), dynamic mechanical analysis (DMA), X-ray diffraction (XRD) analysis, and decay resistance of Scots pine sapwood against white and brown rot fungi as a function of reaction catalysts such as citric acid (CA) and succinic acid (SA). These poly(carboxylic acid)s were selected with the aim of forming highly reactive cyclic anhydride intermediates to facilitate the creation of stable ester bonds between the available hydroxyl groups in both humins and wood polymers,^13^ thus improving the water-related properties of huminated wood.

## Results and Discussion

### Weight and Dimensional Changes

**Figure 1:**
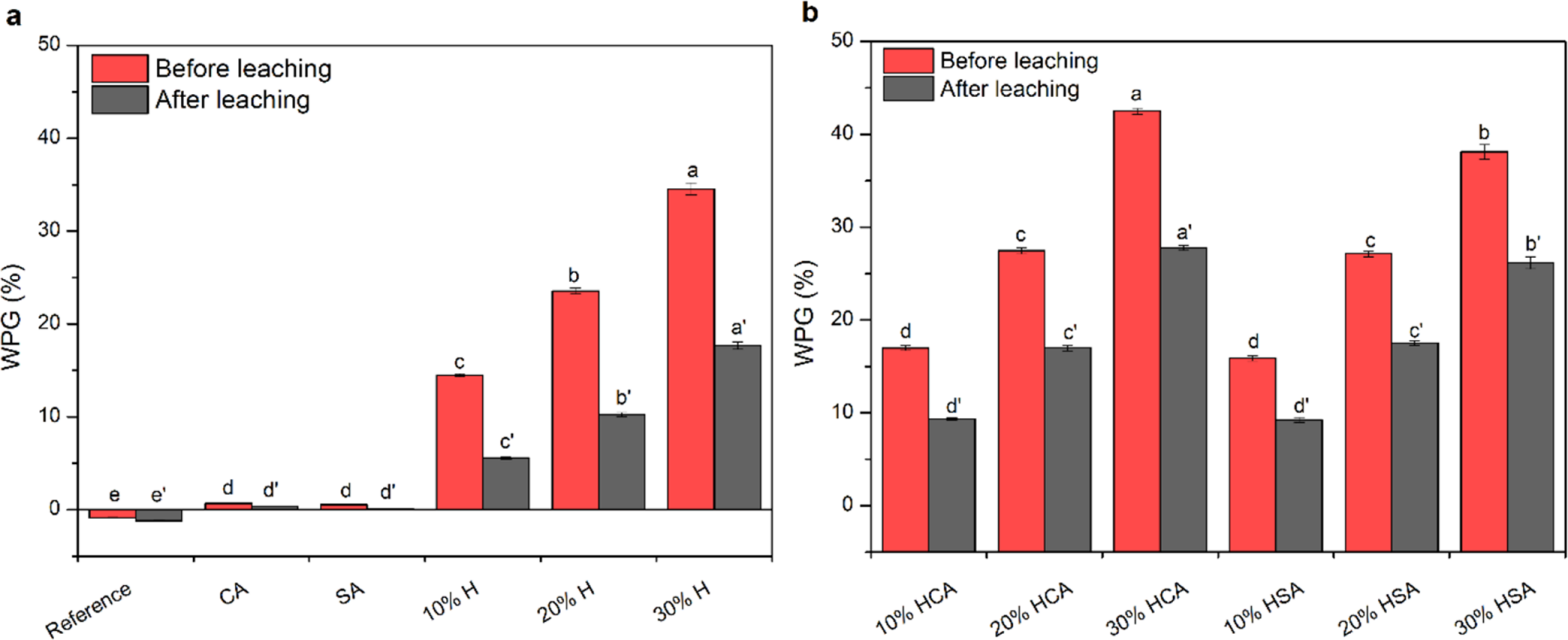
WPG of the reference, catalyst, and sole humin-modified (a) and humin-catalyst-modified (b) samples before and after leaching. Error bars indicate standard error (SE; *n* = 12), and different letters above the bars denote significant differences (*p* < 0.05) in each subfigure.

The weight percentage gain (WPG) of Scots pine sapwood was considerably increased by humination modification and linearly increased with increasing humin concentrations from 10 to 30% (Figure 1). While the modification of the samples with sole catalysts (CA and SA) led to marginal weight changes, humination in the presence of catalysts resulted in a similar trend as sole huminated samples but considerable higher WPG values at identical concentrations. The differences in WPGs were statistically significant (analysis of variance (ANOVA), α = 0.05). Similar trends were observed in the WPG values after 7 days of the leaching test, although the test led to an apparent weight reduction. The WPG reduction in the modified samples containing the catalyst ranged from 45 to 34% in HCA samples and from 41 to 31% in HSA samples, with higher reduction values in lower concentrations of humin. The sole huminated samples, however, illustrated a more apparent WPG reduction of 61, 56, and 48% in the samples modified at 10, 20, and 30% of humin, respectively. The highest WPGs after the leaching tests were obtained in the samples modified with 30% HCA and HSA, which were 27.7 and 26.1%, respectively. This might be due to the formation of water-stable humin-based matrices in the wood cell in the presence of acidic catalysts.

**Figure 2:**
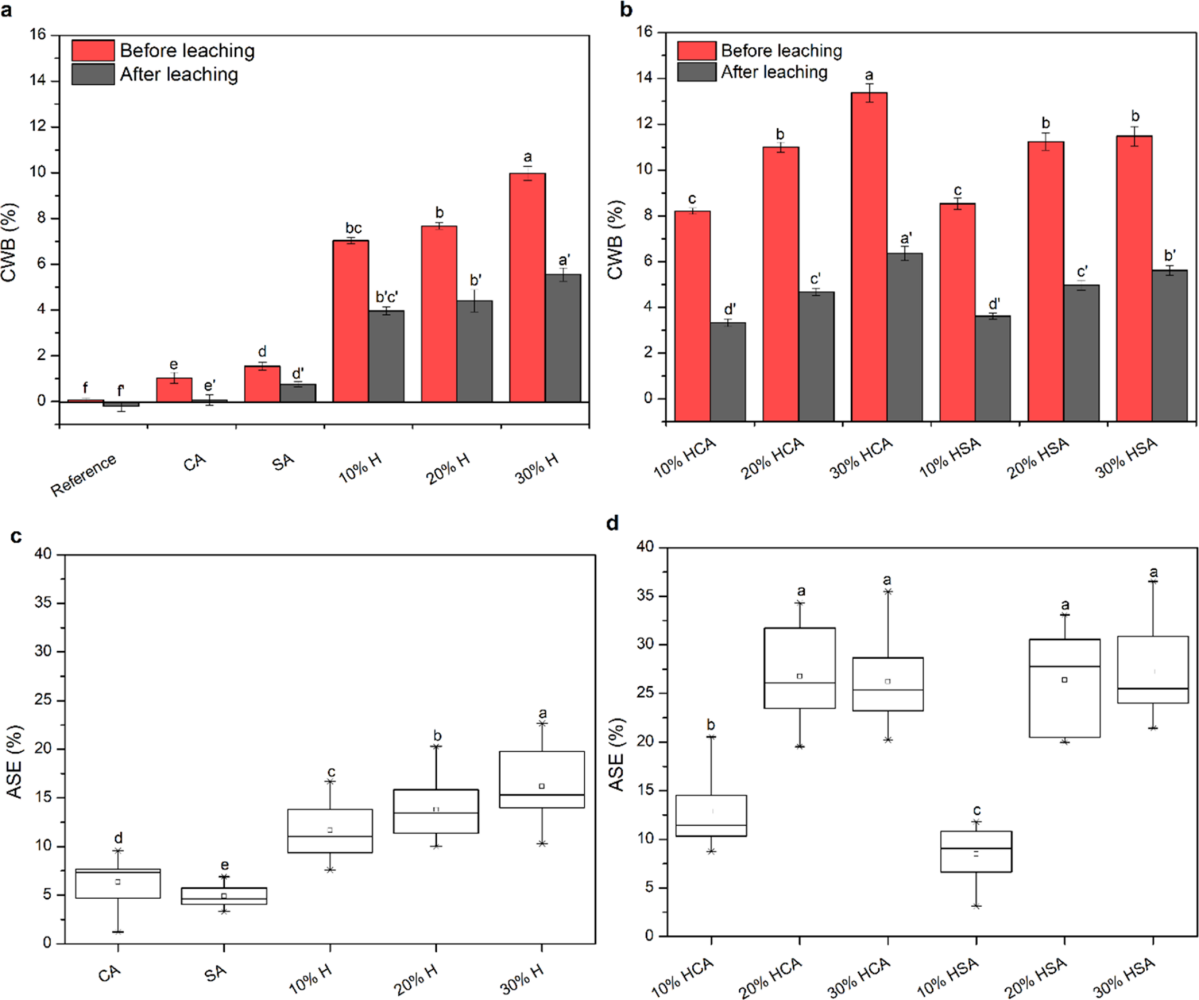
Cell wall bulking (a, b) and ASE (c, d) of Scots pine-modified samples. Error bars indicate standard error (SE; *n* = 12), and different letters above the bars denote significant differences (*p* < 0.05).

The dimensional stability of the Scots pine sapwood samples was highly influenced by the different modification systems. As shown in Figure 2a, the modification of samples with sole CA and SA resulted in a slight increase in the cell wall bulking (CWB), but it almost disappeared after leaching. In contrast, the humination modification caused a more substantial effect on the CWB. The bulking values were linearly increased with the increasing concentration level of humins with no obvious differences between the formulas containing CA and SA (Figure 2b). The CWB values were highly influenced by the leaching test, with a remarkable reduction. Before leaching, the huminated samples containing catalysts showed higher CWB than the ones modified with sole humin at similar concentration levels, but the values became almost comparable after leaching. The CWB refers to an irreversible state in the cell wall where the free spaces between the wood polymers are filled with the cured chemicals, and thus wood is positioned as permanently swollen.^14^ The antiswelling efficiency (ASE) also followed an almost similar pattern with a marginal effect in the samples modified with CA and SA and notably increased at higher concentrations of humins (Figure 2c,d). The addition of the catalysts clearly enhanced the ASE values with a major effect at 20 and 30% humin levels. The latter samples showed that the ASE ranged between 25 and 28%, which was significantly higher than the samples modified with sole humins or catalysts. The ASE values of CA- and SA-modified samples were, respectively, 6.3 and 4.8%. It is assumed that the strong increase in ASE values is due to the decrease of space for water molecules to accommodate within the cell wall matrix as a result of CWB. Blockage of the OH groups of wood polymers after the modification might be an additional reason.^1^ Similar ASE values were reported by Vukusic et al.^15^ for dimethylol-4,5-dihydroxy-ethylene-urea (DMDHEU)-modified beech wood.

### Bending Properties

**Figure 3:**
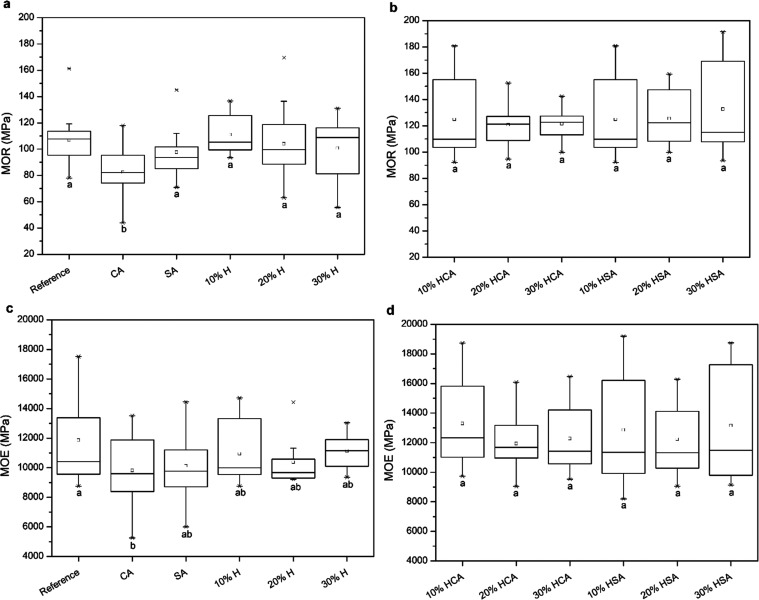
Modulus of rupture (MOR) (a, b) and modulus of elasticity (MOE) (c, d) of the unmodified reference and modified wood samples with humins and catalysts. Different letters below the boxes denote significant differences (*p* < 0.05).

The bending properties of unmodified and modified Scots pine samples were determined in static and dynamic modes. The static bending evaluated the modulus of elasticity (MOE) and the modulus of rupture (MOR), while the dynamic test studied the storage modulus (*G*′) and tan δ as a function of elevated temperatures. The MOR of the samples modified with sole humins slightly decreased with increasing humin concentrations from 10 to 20% and then was comparable to unmodified reference samples at 30% humins (Figure 3a). The differences were statistically insignificant (ANOVA, α = 0.05). Modification with CA and SA resulted in the respective MOR values of 82.4 and 97.6 MPa, which are considerably lower than the reference samples of 106.7 MPa. The differences in the MOR were statistically significant in the case of CA-modified samples according to the ANOVA test using Duncan’s multiple range test comparison at α = 0.05. Humination of the samples in the presence of catalysts, however, improved the MOR with a slight increase in the mean values with increasing humin concentrations in comparison with the unmodified reference samples, although the differences were only statistically significant in the samples modified with 30% HSA that showed the MOR of 132.6 MPa (Figure 3b). An identical behavior was observed in MOE results, in which, compared with the reference samples, the ones modified with catalysts showed lower mean values, no major differences detected by sole humins, and slightly higher values obtained in the huminated samples containing catalysts (Figure 3c,d). As reported previously,^16−18^ the reduction of the MOR and MOE of CA- and SA-modified samples could be attributed to the degradation of wood polymers under acidic conditions. In contrast, humination modification seemed to form matrices in the wood cell that not only interfered with the equal distribution of the applied mechanical stress but also, to some extent, contributed to the strength improvement at higher humin concentrations. Mija et al.^19^ previously reported a similar effect in increasing the Young’s modulus and tensile strength of polyfurfuryl alcohol composites by the addition of humins.

**Figure 4:**
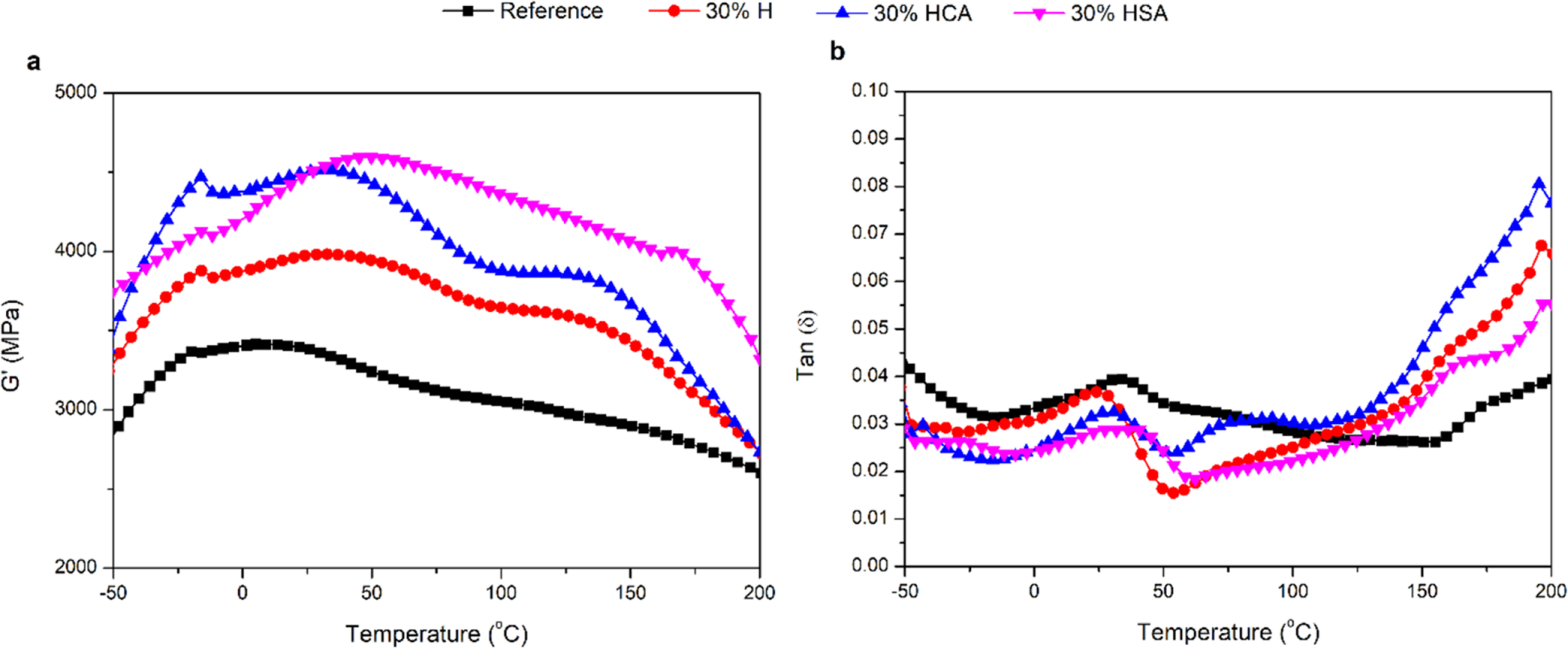
Storage modulus (a) and tan δ (b) of the reference and 30% H-, 30% HCA-, and 30% HSA-modified wood samples.

Figure 4a,b shows the storage modulus (*G*′) and tan δ of wood samples modified with 30% H, 30% HCA, and 30% HSA, as assessed with DMA. The huminated samples showed a considerably higher storage modulus than the unmodified ones in the temperature range from −50 to 200 °C. The storage modulus of the samples slightly increased with increasing temperature from −50 °C, became almost constant at around 50 °C, and then decreased. The unmodified wood showed a slight and gradual reduction in the storage modulus with increasing temperature from 50 °C but a sharp reduction slope was detected in huminated samples. This phenomenon was also reported previously in huminated samples and explained as an effect of temperature on enhancing the polymer mobility,^12^ thus resulting in less rigid matrices at the higher temperature. The tan δ curves showed two relaxation processes at around 45 and 150 °C, which were labeled as β and α. These relaxation temperatures may change depending on the amount of moisture in the wood structure; the higher moisture content (MC) results in lower relaxation temperatures.^20^ The huminated samples exhibited temperatures in the β region lower than that of the reference one. It should be noted that while lignin and the polysaccharides are both capable of exhibiting β relaxations for brief segments of their respective polymer chains, it can be challenging to determine which polymer is responsible for the β-peaks seen in reference wood.^21^ By increasing the temperature, in the α relaxation phase, the values increased compared to the reference samples. The α relaxation process is assigned to the micro-Brownian motions of wood cell-wall polymers in the noncrystalline region.^22^ When a polymer chain changes from a glassy to a viscous state, micro-Brownian movements occur within the chain that is related to the α-peak or glass transition temperature.^22^ Moreover, the higher α relaxation of the huminated samples than the reference one may also be related to the loss of hemicellulose during wood modification and an increase in the hydrophobicity of humin-modified wood.^21^ This may have been a glass transition zone since it was accompanied by a reduction in the storage modulus. The DMA results confirmed that the thermomechanical properties of wood are considerably altered by humination modification. More information regarding the storage modulus and tan δ of other modified samples can be found in the Supporting Information.

### Thermogravimetric Analysis (TGA)

**Figure 5:**
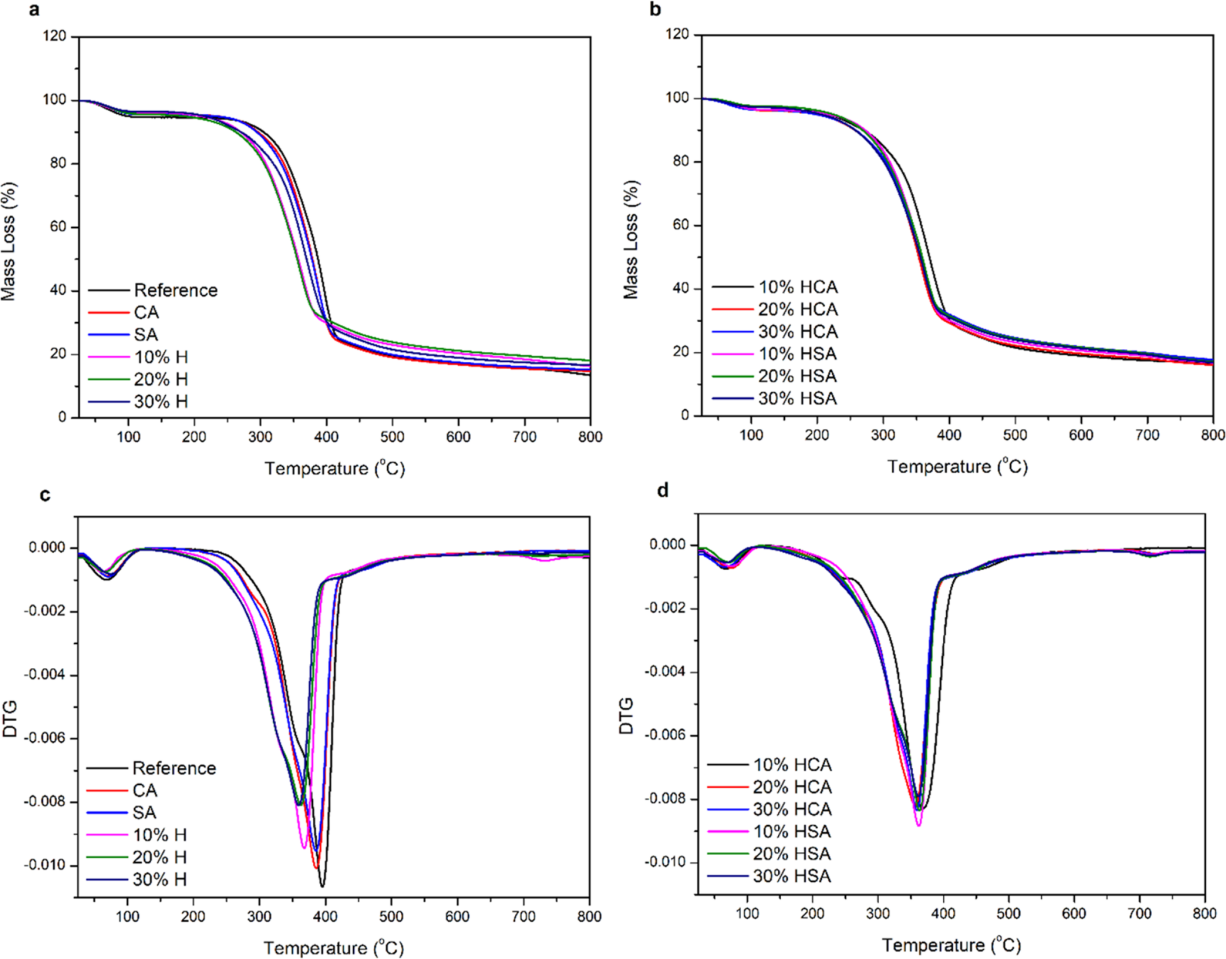
TGA (a, b) and DTG (c, d) curves for the reference and modified wood samples with humins and/or catalysts.

The thermal stabilities and decomposition temperature of modified wood samples were studied by thermogravimetric (TG) and derivative thermogravimetric (DTG) analysis, and the results were compared with the unmodified reference samples (Figure 5). As expected, increasing the temperature facilitated the decomposition of the samples and different degradation patterns were observed as a result of various modification solutions. The initial mass loss was observed at a temperature below 100 °C, which is reflected as Tmax_1_ in Table 1, and it is mostly related to the evaporation of water or other polar solvents with low boiling temperatures as well as water from the incomplete polymerization and degradation of unreacted low-molecular-weight compounds with lower thermal decomposition temperatures.^23,24^ The DTG curves show a shoulder at ∼350 °C in reference samples, which was shifted to lower temperatures in the sole huminated samples and almost disappeared in the ones modified in the presence of CA and SA catalysts. Kim et al.^25^ reported that the depolymerization of hemicellulose takes place between 180 and 350 °C, while random cleavage of the glycosidic bond of cellulose, mainly in the amorphous region, between 250 and 500 °C might also be expected. The major mass losses occurred at temperatures above 350 °C (Tmax_2_). The huminated samples showed a Tmax_2_ of ∼30 °C that was lower than that of the unmodified reference samples. Except for the samples modified with 20% HCA, the Tmax_2_ decreased with increasing the humin content. This might be due to the changes in the crystalline structure of the samples after modification, as shown in Table 1. Interestingly, almost all modified samples showed a higher residual mass (RS) than the unmodified reference sample, and except for 20% HSA- and 20% HCA-modified samples, the RS values also increased with increasing humin content. This might be attributed to the catalytic effect of modification agents in the formation of the thermal stable layer, like a char-shaped one, in the modified samples through chemical cross-linking with wood polymers, which could protect the wood polymers from degrading at higher temperatures,^26^ thus eventually resulting in the high RS. Similar results were reported previously in humin-based composites with cellulosic fibers and jute,^23,27^ and the authors discussed that the slow carbonization rate of humins might be the reason for the high RS.

**Table 1:** Thermal Degradation Properties of Reference and Modified Wood Samples

samples	Tmax_1_ (°C)	Tmax_2_ (°C)	residual mass (%) (800 °C)
reference	68.16	395.17	13.45
CA	69.83	385.83	14.79
SA	70.16	385.50	15.23
10% H	61.66	367.83	12.90
20% H	65.83	362.66	16.31
30% H	74.83	359.33	18.13
10% HCA	67.16	368.83	16.67
20% HCA	76.16	356.50	16.07
30% HCA	64.83	359.16	17.73
10% HSA	74.83	362.00	16.75
20% HSA	72.50	363.16	17.08
30% HSA	70.83	361.50	16.82

### XRD Analysis

**Figure 6:**
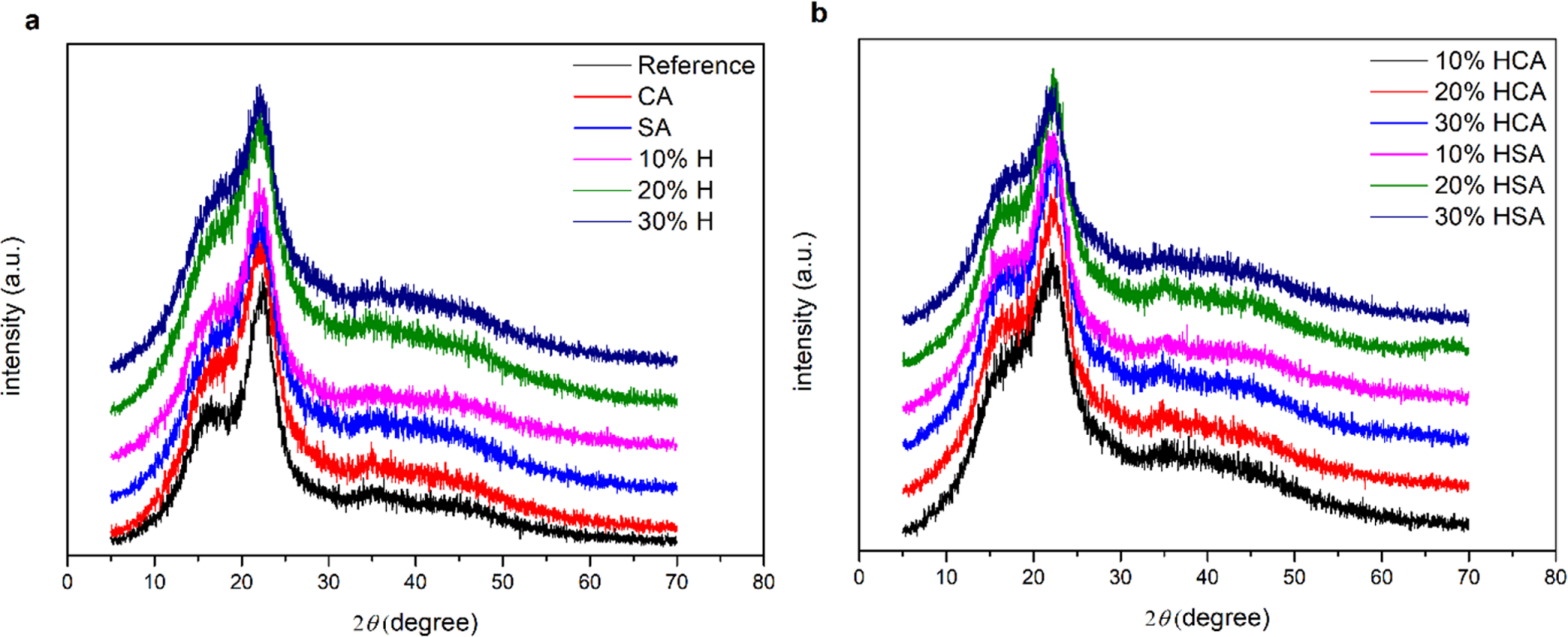
X-ray diffractograms from the reference (a) and modified (b) samples with humins and catalysts.

The crystalline structure and degree of crystallinity of the unmodified and modified wood samples with CA, SA, and different concentrations of humins are shown in Figure 6a,b and Table 2. CrI denotes the crystalline structure, *L* represents the cellulose size, I200 corresponds to the maximum lattice diffraction peak, and Iam signifies the intensity scattered by the amorphous part of the sample.^28^ After modification of the wood samples with humins and catalysts, the amorphous structure of the modified samples slightly increased and the crystalline structure decreased. The differences at the peaks between 15, 25, and 33° corresponded to the I200 and Iam diffraction planes of crystalline cellulose.^29,30^ Both the crystalline index and the crystal size after the modification of the samples decreased. This was correlated with the concentration of humins and the presence of acid catalysts in the modification solution, which might be related to the acid hydrolysis of the cellulose chain.^31^

**Table 2:** Band Positions and the Calculated Parameters for the Crystallinity of the Reference and Modified Wood Samples

sample code	I200	Iam	CrI (%)	*L* (200) (nm)
reference	22.37	18.35	57.09	8.13
CA	21.95	18.01	51.90	8.04
SA	22.05	17.46	48.29	8.19
10% H	22.02	18.64	50.36	8.45
20% H	22.08	18.06	42.66	7.62
30% H	22.05	17.43	41.62	7.59
10% HCA	21.92	17.82	40.36	7.54
20% HCA	22.45	18.30	49.53	7.65
30% HCA	22.26	18.30	42.68	7.82
10% HSA	21.50	19.01	50.94	8.32
20% HSA	22.24	18.16	52.87	8.29
30% HSA	22.55	17.32	42.68	7.55

### Biological Durability

**Figure 7:**
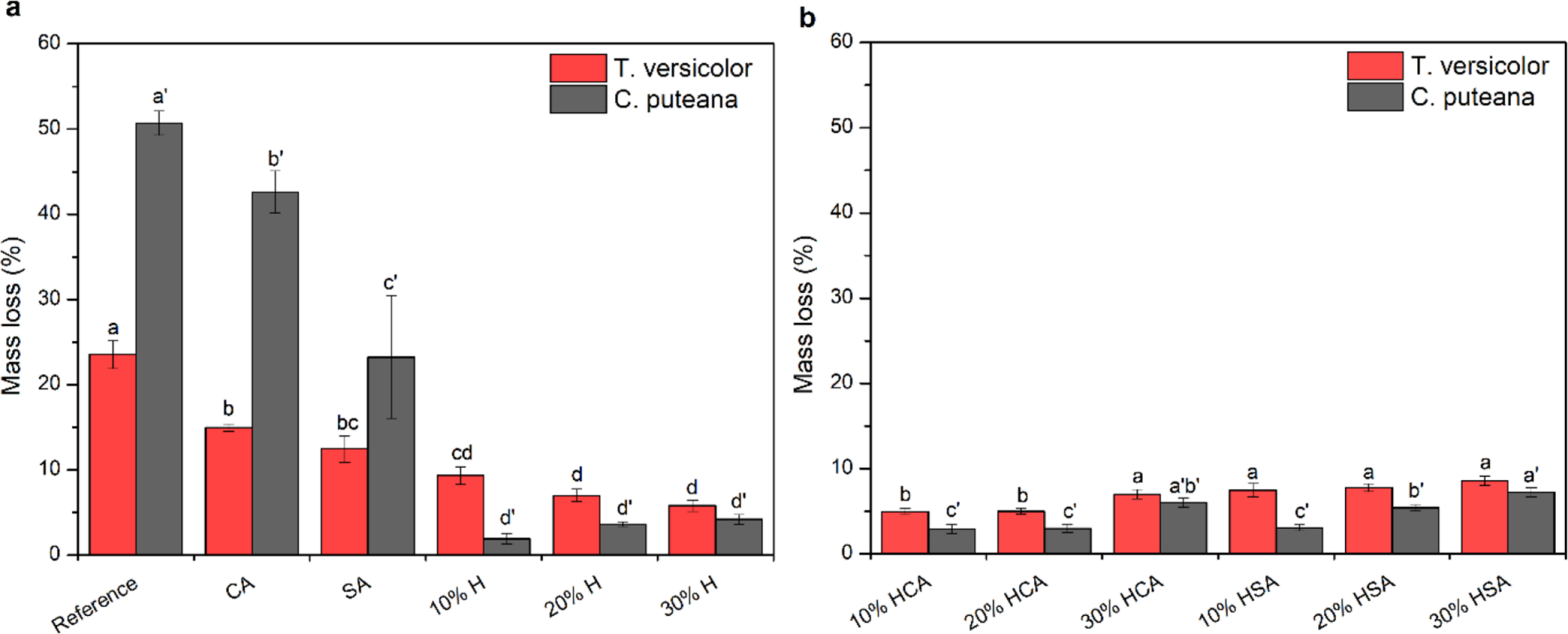
Comparisons of mass loss values of the reference (a) and modified (b) samples after exposure to *T. versicolor* (white rot) and *C. puteana* (brown rot) fungi. Error bars indicate standard error (SE; *n* = 12), and different letters above the bars denote significant differences (*p* < 0.05).

The mass losses of the reference and modified wood samples against the white and brown rot fungi are presented in Figure 7. The tests were deemed valid since the mass losses of the reference samples exposed to fungi types were above 20%, which is the threshold commonly used to indicate sufficient fungal activity for both white and brown rot fungi, as per EN 113-1.^32^ The reference samples exposed to *Coniophora puteana* (brown rot) and *Trametes versicolor* (white rot) showed significant mass losses of 50.7 and 23.5%, respectively. These major mass losses were followed by those of the CA- and SA-modified samples. However, humination modification remarkably improved the decay resistance. For the brown rot test, the lowest mass loss of 1.9% was obtained at 10% humination, while the other huminated samples showed slightly higher mass losses. The differences between huminated samples were statistically insignificant, except for 30% HCA and 30% HAS, which illustrated significantly higher mass losses as compared with 10% H. In contrast, the resistance of huminated samples to the white rot fungi was influenced by the concentration of humin in the sole huminated samples, where the resistance was significantly increased from 10% (9.3% mass loss) to 30% (5.8% mass loss). The presence of CA further increased the resistance to white rot decay, presenting an average mass loss value of 5% in the 20% HCA-modified samples. The opposite effect was observed in the HSA-modified samples, resulting in an increase in the average mass loss with an increase in the humin content, even though the differences were statistically insignificant. The decay resistance of the huminated samples is considerably higher than DMDHEU,^33^ acetylation,^34^ and glutaraldehyde^35^ modifications and almost identical to furfurylated wood^36^ at comparable or even higher WPGs that were reported previously and can be due to several factors. The high mass retention of huminated samples compared to the CA- and SA-modified samples and the reference ones might be related to their apparent effect on reducing water absorption due to the cell wall bulking, as shown in Figure 2, which limits the diffusion coefficients for transport of substances in the cell wall, which inhibits the ingress of diffusible agents from fungi.^2^ The differences between the mass loss of different huminated samples exposed to brown rot and white rot fungi can be explained by the effect of modification on the wood polymers. The marginal mass loss of the huminated wood exposed to the brown rot fungus might be related to the creation of covalent linkages between the accessible functional groups in cellulose and hemicellulose and humins and/or the physical masking of these polymers through film formation. These effects were, to a minor extent, offset with increasing the humin concentration from 10 to 30% and also the presence of the catalyst by altering the crystalline structure of wood, as shown in Table 2. The degradation resistance of the samples against the white rot fungi at the higher concentration of humin, e.g., in 30% H-modified samples, and in the presence of CA might be related to the possible interaction between the humin-based solution and lignin, which protected lignin from biological degradation. A similar pattern was reported previously in protective lignin again photodegradation.^11^ Overall, it can be concluded that the property improvement of huminated wood might be due to both cell wall bulking and the chemical interaction with wood polymers.

## Conclusions

This study revealed that the humination modification considerably improved the water-related properties and resistance to white and brown rot fungi in Scots pine sapwood. The humination modification altered the static mechanical properties, thermal degradation stability, and crystalline structure and substantially enhanced the storage modulus of the wood samples. We found that the properties of modified wood were also influenced by the presence of catalysts and the concentration of humins, where the most convincing results were obtained from the samples modified at a 20% humin level and the CA catalyst (20% HCA). The cell wall bulking and interaction between humin-based solutions and wood polymers were detected as the major reasons for such behaviors in wood material properties, and thus, it can be claimed that the humination modification cannot be placed in the classical modification categories as a purely active or passive wood modification, while it can be called a semiactive modification process.

## Experimental Section

### Materials

Sapwood boards from Scots pine (*Pinus sylvestris* L.) were obtained from a local sawmill in Kronoberg, Sweden, with an average density of 490 kg m^–3^. For testing, samples without knots or visible defects were selected from these boards. The sapwood lumber was kiln-dried to approximately 12% moisture content (MC) before use.

Humins were kindly supplied by Avantium Renewable Polymers B.V. (Amsterdam, The Netherlands) as a byproduct of the fructose and glucose dehydration process. Citric acid (CA, 98%) and succinic acid (SA, 99%) were obtained from Sigma-Aldrich (St. Louis, MI) and used as catalysts.

### Humination

Due to the high viscosity of tarlike humin, it was initially heated at 60 °C, and then it was added to a three-neck flask partially submerged in an oil bath containing preheated water (at 60 °C) at a water/humin mass ratio of 1:1. The mixture was stirred for 6 h at 60 °C at 2000 rpm for 10 min using an Ultra-Turrax (IKA T18D, Germany). Afterward, the mixture was cooled at room temperature until two distinct phases appeared. The water-soluble fraction with a solid content of 30% was then collected and further diluted to prepare humin solutions at 20 and 10% concentrations. Additional modification solutions were prepared using 1.5% (weight solid/weight solid humin) CA and SA as reaction catalysts. Detailed information of the modification solutions is shown in Table 3. The humination process was carried out by vacuum-impregnating (370 mbar) oven-dried (103 °C/24 h) wood samples at room temperature for 4 h. After impregnation, the samples were removed from the solutions, followed by blotting off the excess material from the surface. The samples were kept at room temperature for 24 h and then placed in an oven at 40 °C. The curing process started by gradually increasing the temperature from 40 to 103 °C with a ramp of 20 °C per 24 h and then directly to 140 °C for 10 h. Prior to testing, the samples were kept at 20 °C and 65% relative humidity (RH) for a duration of 14 days.

**Table 3:** Details on the Modification Solutions

no.	sample code	modification agent	catalyst	pH
1	reference	water		6.8
2	CA	water	CA	5.3
3	SA	water	SA	5.6
4	10% H	10% humin		5.8
5	20% H	20% humin		5.6
6	30% H	30% humin		5.3
7	10% HCA	10% humin	CA	5.2
8	20% HCA	20% humin	CA	4.9
9	30% HCA	30% humin	CA	4.8
10	10% HAS	10% humin	SA	5.2
11	20% HAS	20% humin	SA	5.0
12	30% HAS	30% humin	SA	5.0

### Weight and Dimensional Changes

The weight changes of 12 samples (*n* = 12) measuring 25 × 25 × 10 mm^–3^ (*R* × *T* × *L*) were determined after humination and 7 days of leaching. The latter was performed by reaching a stable pH level. The volumetric changes of the samples were recorded due to the leaching process to evaluate the cell wall bulking (CWB), as described previously.^1,5^ Then, samples were exposed to two cycles of water impregnation and drying, according to Hosseinpourpia et al.,^1^ to determine the antiswelling efficiency (ASE).

### Static and Dynamic Bending Properties

The ASTM D143-14^37^ standard was followed to determine the static three-point bending properties such as the modulus of elasticity (MOE) and the modulus of rupture (MOR) using MTS-10kN universal testing. Ten replications of samples with the dimensions of 10 × 10 × 180 mm^–3^ (*R* × *T* × *L*) were prepared per modification condition.

Dynamic mechanical analysis (DMA) was performed using GABO EPLEXOR DMA 800 (GABO qualimeter Testanlagen GmbH, Germany), as described previously by Hosseinpourpia et al.^38^ The modified and unmodified samples measuring 10 × 5 × 30 mm^–3^ (*R* × *T* × *L*) were scanned over a temperature range of −50 to 200 °C. The frequency of the oscillation was fixed at 1 Hz, and the temperature was ramped at 2 °C min^–1^.

### Thermogravimetric Analysis (TGA)

The thermal degradation stability of samples was evaluated, according to Hosseinpourpia et al.,^38^ using a Mettler-Toledo 2-HT thermogravimetric analyzer (Mettler-Toledo, Schwerzenbach, Switzerland). Under a nitrogen atmosphere, the reference and modified samples underwent thermogravimetric analysis (TGA). About 10 mg of each sample was placed in a 70 μL ceramic test pan and heated from 25 to 800 °C at a rate of 10 °C min^–1^.

### X-ray Diffraction (XRD) Analysis

The crystalline structure of modified and unmodified milled samples was analyzed using a Philips X’Pert PRO automatic diffractometer operating in a θ–θ configuration with an operating voltage of 40 kV and a current of 40 mA. The use of a secondary monochromator with Cu Kα radiation (λ = 1.5418 Å) and a PIXcel solid-state detector (active length in 2θ = 3.347°) allowed for the observation of diffracted X-rays. A fixed divergence/antique scattering slit was employed to maintain a constant sample volume during radiation exposure. According to Ghavidel et al.,^30^ the crystallinity index (CrI) was assessed from the crystalline and amorphous peak intensities and the apparent crystallite size was estimated using the Scherrer equation.

### Resistance to Basidiomycetes

The biological durability of unmodified and modified samples was evaluated by exposure to *Trametes versicolor* (white rot) and *C. puteana* (brown rot) fungi according to XP CEN/TS 15083-1.^39^ As detailed by Ahmed et al.,^40^ 12 mini-block specimens of 10 × 5 × 30 mm^–3^ (*R* × *T* × *L*) per sample type were incubated in malt agar media at 22 °C and 65% RH for 16 weeks. The validity of the tests was confirmed by placing European beech wood (*Fagus sylvatica* L.) as a reference in the flasks. After incubation, the samples were removed from the Kolle flasks, cleaned from adhering fungal mycelium, oven-dried, and weighed to assess the mass loss due to fungal decay using the following formula:
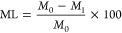
where ML is the mass loss after fungal decay (%), *M*_0_ is the oven-dry mass of the wood samples before incubation (g), and *M*_1_ is the oven-dry weight of the samples after incubation (g).

### Statistical Analysis

Statistical differences between the variants were determined using IBM SPSS version 26 (IBM Corporation, New York). A one-way analysis of variance (ANOVA) was conducted with a significance level of 0.05, followed by Duncan’s multiple range test to analyze significantly different samples.

## Supporting Information Available

The storage modulus and tan δ for all of the reference and modified samples treated with various modification solutions (PDF)
