# Supplementary material for: Humination Modification: A Green Approach to Improve the Material Properties of Scots Pine (Pinus sylvestris L.) Sapwood
Source: ACS Omega. 2025 Jan 15;10(3):2996–3005. doi: 10.1021/acsomega.4c09540 (PMC11780435; doi:10.1021/acsomega.4c09540)
Supplement: Supplementary file 1 — ao4c09540_si_001.pdf [file ao4c09540_si_001.pdf]

Supporting information

Humination Modification: A Green Approach to Improve the Material Properties of Scots Pine (Pinus sylvestris L.) Sapwood

Amir Ghavidel [a], Arantxa Eceiza [b], Xinfeng Xie [c], Reza Hosseinpourpia [c,d]*

[a.] School of Engineering, University of Northern British Columbia, 499 George St., Prince George V2L1R7, British Columbia, Canada

[b.] Materials + Technologies' Group, Chemical & Environmental Engineering Dep., Polytechnic College of San Sebastian, University of the Basque Country UPV/EHU, Pza. Europa 1, 20018 Donostia-San Sebastián, Spain

[c.] College of Forest Resources and Environmental Science, Michigan Technological University, Houghton, Michigan 49931, United States

[d.] Department of Forestry and Wood Technology, Linnaeus University, Lückligs Plats 1, 35195 Växjö, Sweden

* Correspondence: reza.hosseinpourpia@lnu.se; Tel.: +46-470 70 80 74

Figure S1 and S2 perecent the storage modulus and tan δ for all the reference and modified samples treated with different modification solutions.

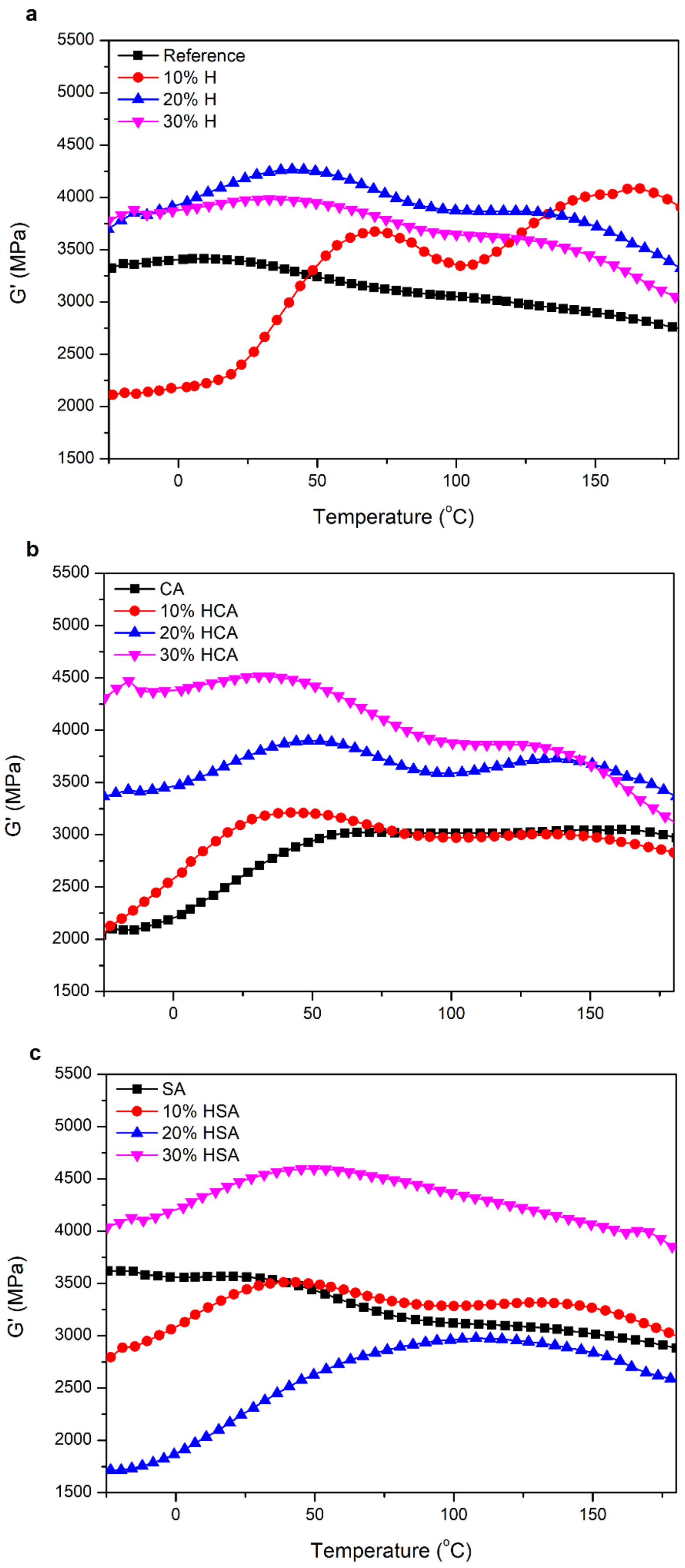
a
Reference
10% H
20% H
30% H
G' (MPa)
5500
5000
4500
4000
3500
3000
2500
2000
1500
0
50
100
150
Temperature (°C)
b
CA
10% HCA
20% HCA
30% HCA
G' (MPa)
c
SA
10% HSA
20% HSA
30% HSA
G' (MPa)
Temperature (°C)

Figure S1. Storage modulus (a) reference and solo humins, (b) citric acid-only and different cuncentration of HCA, (c) succinic acid-only and different cuncentration of HAS.

a

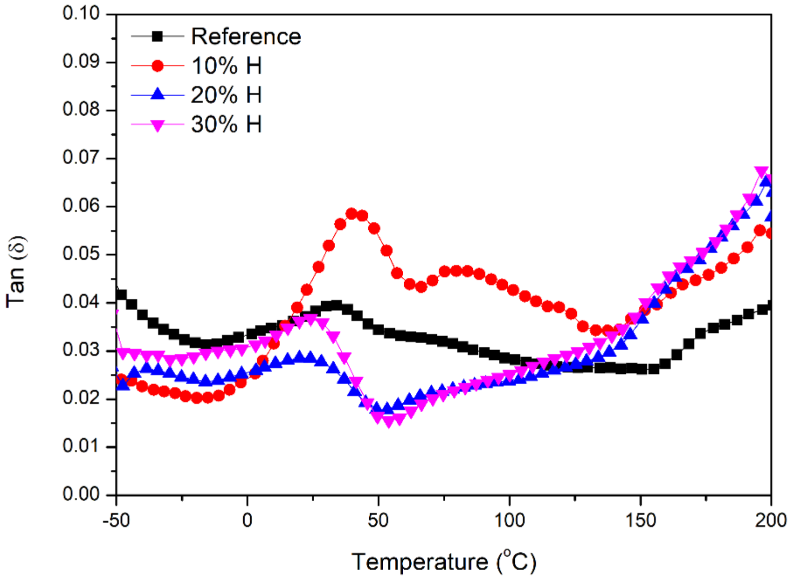

Reference
10% H
20% H
30% H
Tan (δ)
Temperature (°C)

b

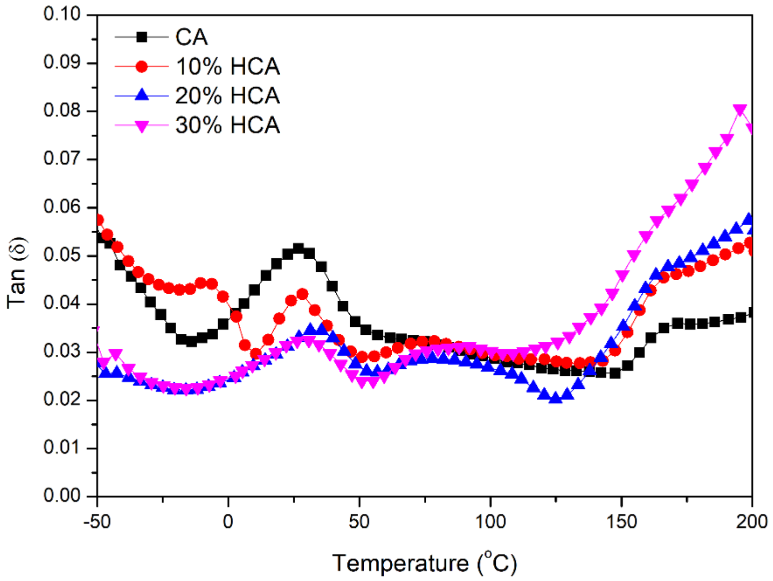

CA
10% HCA
20% HCA
30% HCA
Tan (δ)
Temperature (°C)

c

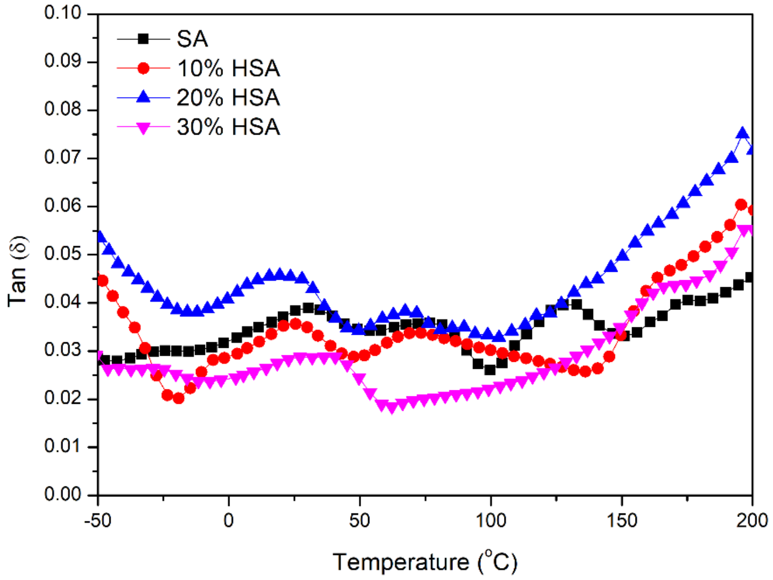

SA
10% HSA
20% HSA
30% HSA
Tan (δ)
Temperature (°C)

Figure S2. tan δ (a) reference and solo humins, (b) citric acid-only and different cuncentration of HCA, (c) succinic acid-only and different cuncentration of HAS.
